# Characterization and comparative analysis of HMW glutenin *1Ay *alleles with differential expressions

**DOI:** 10.1186/1471-2229-9-16

**Published:** 2009-02-06

**Authors:** Qian-Tao Jiang, Yu-Ming Wei, Feng Wang, Ji-Rui Wang, Ze-Hong Yan, You-Liang Zheng

**Affiliations:** 1 Triticeae Research Institute, Sichuan Agricultural University, Ya'an, Sichuan, 625014, PR China; 2 Key Laboratory of Crop Genetic Resources and Improvement, Ministry of Education, Sichuan Agricultural University, Ya'an, Sichuan, 625014, PR China

## Abstract

**Background:**

High-molecular-weight glutenin subunits (HMW-GSs) have been considered as most important seed storage proteins for wheat flour quality. 1Ay subunits are of great interest because they are always silent in common wheat. The presence of expressed 1Ay subunits in diploid and tetraploid wheat genotypes makes it possible to investigate molecular information of active *1Ay *genes.

**Results:**

We identified 1Ay subunits with different electrophoretic mobility from 141 accessions of diploid and tetraploid wheats, and obtained the complete ORFs and 5' flanking sequences of *1Ay *genes including 6 active and 3 inactive ones. Furthermore, the 5' flanking sequences were characterized from 23 wild diploid species of Triticeae. All 6 active *1Ay *possess a typical HMW-GS primary structure and some novel characteristics. The conserved cysteine residue within the repetitive domain of y-type subunits was replaced by phenylalanine residue in subunits of 1Ay (Tu-e1), 1Ay (Tu-e2), 1Ay (Ta-e2) and 1Ay (Td-e). Particularly, *1Ay (Ta-e3) *has an unusual large molecular weight of 2202 bp and was one of the known largest y-type HMW-GSs. The translations of *1Ay (Tu-s)*, *1Ay (Ta-s) *and *1Ay (Td-s) *were disrupted by premature stop codons in their coding regions. The 5' flanking sequences of active and inactive *1Ay *genes differ in a few base substitutions and insertions or deletions. The 85 bp deletions have been found in promoter regions of all *1Ay *genes and the corresponding positions of 6 species from *Aegilops *and *Hordeum*.

**Conclusion:**

The possession of larger molecular weight and fewer conserved cysteine residues are unique structural features of *1Ay *genes; it would be interested to express them in bread wheat and further to examine their impact to processing quality of wheat. The *1Ay *genes from *T*. *urartu *are closer to the genes from *T*.* turgidum dicoccon *and *T*.* aestivum*, than those from *T*. *monococcum aegilopoides*. The 85 bp deletion and some variations in the 5'flanking region, have not interrupted expression of *1Ay *genes, whereas the defects in the coding regions could be responsible to the silence of the *1Ay *genes. Some mutational events in more distant distal promoter regions are also possible causes for the inactivation of *1Ay *genes.

## Background

In wheat and its relatives, seed storage proteins are mainly composed of glutenins and gliadins [1]. High-molecular-weight glutenin subunits (HMW-GSs) are important storage proteins in endosperm of wheat and its related species [1]. HMW-GSs play a key role in determining wheat gluten and dough elasticity which promote the formation of the larger glutenin polymer [2,3]. The allelic variation in HMW-GS compositions has been reported to account for up to 70% of the variation in bread making quality among European wheats, even though they only account for about 10% of seed storage proteins [2,4]. Therefore, HMW-GS genes are important and useful in molecular modification to improve the wheat grain quality.

HMW-GSs are encoded by the *Glu-1 *loci on the long arms of chromosomes 1A, 1B and 1D, and each locus consists of 2 tightly linked genes encoding an x-type and a y-type subunit, respectively. Theoretically, hexaploid wheat could contain 6 different HMW-GSs, however, gene silence resulted in variation of HMW-GS number: from 3 to 5 subunits in hexaploid bread wheat and from 1 to 3 subunits in durum wheat [5,6]. Among all 6 HMW-GSs, 1Dx, 1Dy and 1Bx are always active, and 1Ax and 1By sometimes appear silent. In hexaploid wheat, the gene encoding 1Ay subunit is always silent. However, 1Ay subunits have been reported in some diploid and tetraploid wheats [7]. Although the expressed 1Ay subunits in 2 accessions of wheat have been reported [8,9], such subunits have never been confirmed by further molecular characterization. To date, more than 20 HMW-GS alleles have been isolated from wheat and its related species [10-30], and these information has greatly improved our understanding in structure, heredity and expression of HMW-GSs. However, our knowledge on *1Ay *genes is still deficient. The expression of 1Ay subunits in some wild diploid and tetraploid wheats offers an opportunity to isolate and analyze nucleotide sequences of active HMW glutenin *1Ay *genes [7,19,31,32]. *Triticum urartu *(AA, 2n = 14), *Triticum monococcum aegilopoides *(AA, 2n = 14) and *Triticum turgidum dicoccon *(AABB, 2n = 28) are important species possibly involved in the evolution process of hexaploid wheat. These species possess many excellent characteristics such as high content of seed protein and high resistance to stripe rust, scab and stress, which could be potentially employed to improve the agronomic traits of common wheat [33].

In this study, we reported the identification of expressed 1Ay subunits from total 141 accessions of *T*.* urartu*, *T*. *monococcum aegilopoides *and *T*.* turgidum dicoccon*, and the characterization of the coding and promoter region sequences of 6 active and 3 inactive *1Ay *genes. To further understand the control of this allele expression, we also characterized the 5' flanking sequences of y-type HMW-GS genes from 23 wild diploid species of Triticeae. The objectives of this study are: 1) to compare promoter and coding region structures of active and inactive *1Ay *alleles, and further to understand the control of *1Ay *gene expression; 2) to compare the primary structure of 1Ay subunits with other known HMW-GSs and analysis the evolution of *Glu-A1-2 *alleles; 3) to provide the basis of the genetic transformation of active *1Ay *gene to verify their effect on wheat processing quality.

## Results

### SDS-PAGE profiles of HMW-GSs

**Figure 1:**
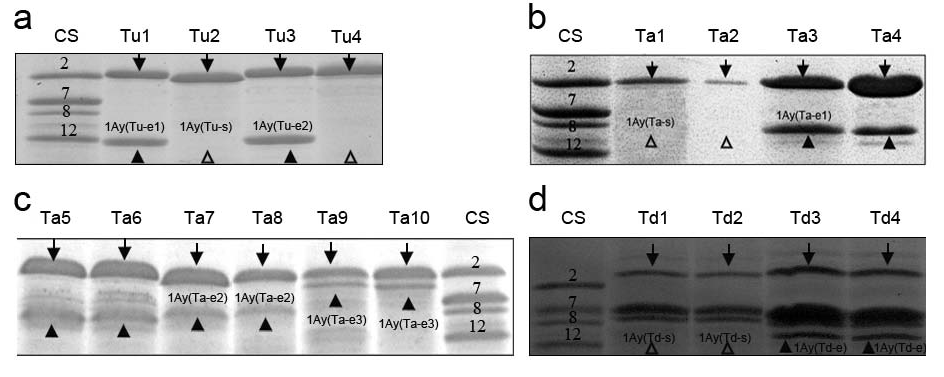
**SDS-PAGE analysis of high-molecular-weight glutenin subunits (HMW-GSs) of diploid and tetraploid wheat species**. **a **Diploid accessions of *T*.* urartu*: (Tu1) PI428309, (Tu2) PI 428308, (Tu3) PI 428318, (Tu4) PI 428310; **b, c**: Diploid accessions of *T*. *monococcum aegilopoides*: (Ta1) PI 427928, (Ta2) PI 427759, (Ta3) PI 428007, (Ta4) PI 427622, (Ta5–6) Citr 17665, (Ta7–8) PI 277123, (Ta9–10) PI 306526; **d: **Tetraploid wheat accessions of *T*. *turgidum dicoccon*: (Td1–2) PI 355475, (Td3–4) PI 355477; CS: Chinese spring. The SDS-PAGE profiles of HWM-GSs showed 1Ay subunits were differentially expressed in some accessions of *T*.* urartu*, *T*. *monococcum aegilopoides *and *T. turgidum dicoccon *while 1Ax subunits were expressed in all accessions (marked by tailed-arrows). The expressed 1Ay subunits were marked by solid and the hollow arrows indicated the area where the absent subunit band might have been.

The SDS-PAGE profiles of HMW-GSs showed that 1Ay subunits were differentially expressed in *T*.* urartu*, *T*. *monococcum aegilopoides *and *T*. t*urgidum dicoccon*, whereas 1Ax subunits were expressed in all accessions of these 3 species (Figure 1). In *T*. *urartu *and *T*. *turgidum dicoccon*, 1Ay subunits displayed an electrophoretic mobility similar to that of 1Dy12 subunit. 1Ay subunits from *T*. *monococcum aegilopoides *migrated slower than those of *T*. *urartu*, showing a similar electrophoretic mobility with 1By8. Interestingly, 1Ay subunit in one accession (PI306526) of *T*.* monococcum aegilopoides *migrated slower than all y-type subunits and 1Bx7. To our knowledge, the y-type HMW-GS with such slower electrophoretic mobility has never been reported, indicating that this subunit might possess a molecular mass larger than other y-type subunits. We also found that for the expression frequency of 1Ay subunits, diploid wheats are higher than tetraploid wheats (Additional file 1).

### Characterization of *1Ay *coding sequences from diploid and tetraploid wheats

**Figure 2:**
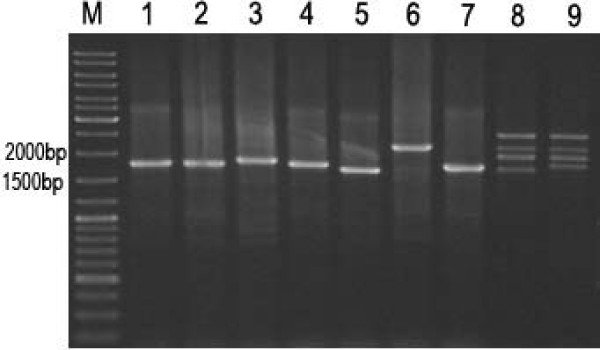
**PCR amplification of HMW-GS ORFs**. Lane1–3: PI 428309, PI 428318, PI 428308 (*T*.* urartu*); lane 4–7: PI 428007, PI 277123, PI 306526, PI 427928 (*T*.* monococcum aegilopoides*) and lane 8 and 9: PI355475, PI355477 (*T*.* turgidum dicoccon*); M is 1 Kb DNA ladder.

In genomic PCR, there is only one amplified fragment in each of *T*.*urartu *and *T*.*monococcum aegilopoides*, whereas 4 fragments were amplified in 2 *T*.*turgidum dicoccon *accessions. The amplified fragments in *T*. *urartu *and *T*.*monococcum aegilopoides *ranged from1800 to 2202 bp (Figure 2). It is close to the size of those typical y-type HMW-GS genes except for the fragment of 2202 bp. In *T*.*turgidum dicoccon *accessions, the molecular weight of fragments is between 1.8 and 2.5 kb (Figure 2). All amplified products were cloned. By terminal sequencing and enzyme digestions, the ORFs representing different *1Ay *alleles were determined. The full length sequences of *1Ay *ORFs were obtained by using the method of nested deletion. The 9 sequences were named as *1Ay (Tu-e1)*, *1Ay (Tu-e2) *and *1Ay (Tu-s) *to represent the ORFs of 1Ay subunits from *T*. *urartu*;*1Ay (Ta-e1)*, *1Ay (Ta-e2)*, *1Ay (Ta-e3) *and *1Ay (Ta-s) *to represent the ORFs of 1Ay subunits from *T*. *monococcum aegilopoides*; and *1Ay (Td-e) *and *1Ay (Td-s) *to represent the ORFs of 1Ay subunits from *T*.*turgidum dicoccon *(the letter e and s represent the expressed and silenced subunits, the numbers represent different alleles.). All sequences were deposited in NCBI database with Genbank accession numbers from:EU984503 to EU984511.

### The primary structures of deduced 1Ay proteins

**Figure 3:**
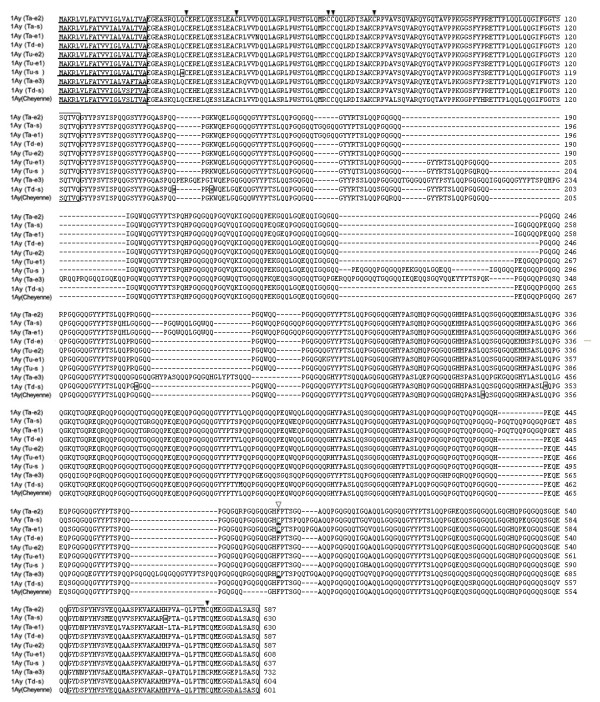
**Comparison of the primary structure of 1Ay subunits from different wheat species. Signal peptide was underlined; N-terminal and C-terminal regions were boxed, respectively**. Conserved cysteine residues were indicated by solid arrows while the substitutions of cysteine residues with phenylalanine residue (F) were marked by hollow arrows. The in-frame stop codons were represented by asterisks and boxed.

After translating the DNA into protein sequences, analysis of amino acid sequence indicated that the ORFs of 6 active *1Ay *genes possess a typical primary structure shared by other published HMW-GSs, although these subunits differ greatly in sizes (Figure 3 and Table 1). Each of these deduced subunits consists of a signal peptide with 21 amino acids (aa), a conserved N-terminal region, a central repetitive domain and a C-terminal region. The N-terminal regions of these 6 subunits contain 104 aa and the C-terminal regions have 42 aa. Central repetitive domains of these subunits are composed of a similar repeat structure to other known y-type subunits. The subunit 1Ay (Ta-e3) is composed of 732 aa, larger than all other known y-type HMW-GSs. The difference between 1Ay (Ta-e3) and other y-type HMW-GSs were entirely due to variations of the number of repeat motifs. Compared to other 1Ay subunits, 13 extra hexapeptides and 5 extra nonapeptides have been inserted into the repetitive domain of 1Ay (Ta-e3), which resulted in 123 aa increases in its molecular mass.

All conserved cysteine residues presented in known HMW-GSs from wheat and its relative grasses were observed in the aa sequences of 1Ay (Ta-e1) and 1Ay (Ta-e3). For 1Ay (Ta-e1) and 1Ay (Ta-e3), the distributions of the 7 cysteine residues are conserved with 5 in N-terminal region, 1 at the end of central repetitive domain and 1 in C-terminal region. However, the conserved cysteine residues at the end of the central repetitive domain of *1Ay (Tu-e1)*, *1Ay (Tu-e2)*, *1Ay (Ta-e2) *and *1Ay (Td-e) *was replaced by phenylalanine residues (Figure 3, Table 1). The translation of the sequence of *1Ay (Tu-s)*, *1Ay (Ta-s) *and *1Ay (Td-s) *were disrupted by in-frame premature stop codons (Figure 3). In the coding sequences of *1Ay (Tu-s) *and *1Ay (Ta-s)*, there is 1 stop codon located in the N-terminal and C-terminal region, respectively; and 4 stop codons were located in the repetitive domain of *1Ay (Td-s)*. If the premature stop codons were ignored, the resulted peptides of 1Ay (Tu-s), 1Ay (Ta-s) and 1Ay (Td-s) would also have typical characteristics of HMW-GSs.

**Table 1:** Summary of primary structure properties of expressed 1Ay subunits compared to those of previously reported y-type subunits.

	Number of amino acid residues				Number of cysteine residues			
		
	N-terminal domain	Repetitive domain	C-terminal domain	Total	N-terminal Domain	Repetitive Domain	C-terminal domain	Total
1Ay (Tu-e1)	104	438	45	597	5	0	1	6
1Ay (Tu-e2)	104	417	45	566	5	0	1	6
1Ay (Ta-e1)	104	461	45	610	5	1	1	7
1Ay (Ta-e2)	104	417	45	566	5	0	1	6
1Ay (Ta-e3)	104	583	45	732	5	1	1	7
1Ay (Td-e)	104	417	45	566	5	0	1	6
1By9	104	535	45	684	5	1	1	7
1Dy10	104	478	45	627	5	1	1	7
1Dy12	104	490	45	639	5	1	1	7

All y-type HMW-GS genes of hexaploid wheat have a conserved cysteine residue at the end of repetitive domain. However, this cysteine residue was found not always present in wild wheats.

### Structural features of the 5' flanking promoter regions of *Glu-A1-2 *alleles and those in 23 Triticeae species

The 5' flanking promoter regions of both active and inactive *1Ay *from diploid and tetraploid wheat species were amplified using the primers P3 and P4. In previous study, regulatory elements (TATA box, complete HMW enhancer, partial HMW enhancer, E motif and N motif) have been identified in the study of promoter activity in wheat endosperm [34,35]. D'Ovidio (1996) previously reported the sequence locations of 5' flanking promoter regions of *1Ay *alleles in *T*. *urartu *to the positions of -595 bp upstream of translational start codon. In this study, we extended the sequences to the positions -845 bp to cover all recognized elements mentioned above. It's more scientific to carry out the promoter comparison using the sequences including all recognized elements. Although comparative analysis of promoter could not directly decide difference in function, it would useful in identification of regulatory elements variations which are relevant to gene function and evolution.

**Figure 4:**
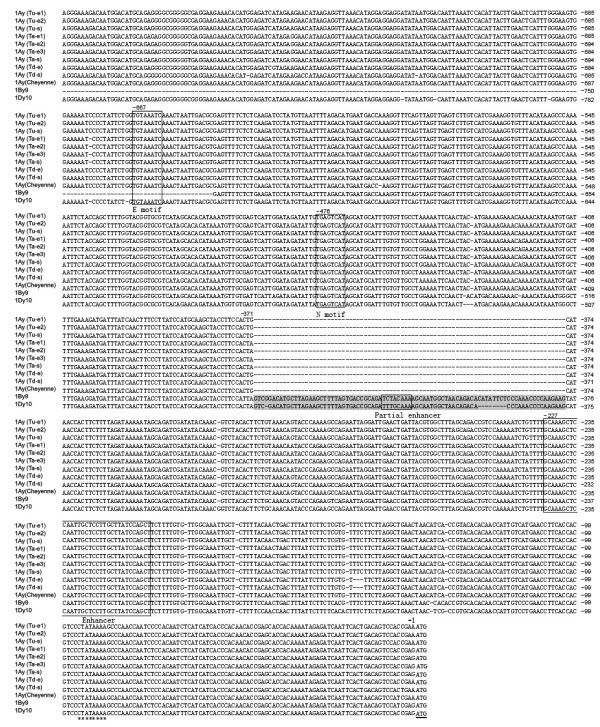
**Comparison of the 5' flanking sequences of 9 *Glu-A1-2 *alleles characterized in this study with those of *Glu-A1-2*, *Glu-B1-2 *and *Glu-D1-2*, represented by *1Ay (Cheyenne)*, *1By9 *and *1Dy10***. The regulatory elements E motif, N motif, partial HMW enhancer and complete HMW enhancer were boxed and labelled, respectively. TATA box was indicated by asterisks; and translational start codon was underlined. This comparison showed that the 85-bp fragment (marked by shadow) was deleted at the 5' flanking sequences of all alleles of *Glu-A1-2*. The 5' flanking sequences of *Glu-A1-2 *alleles from wild diploid, tetraploid and hexaploid wheat species shared high degree of homology.

All characterized promoter regions of *1Ay *were aligned to the homologous regions of *1Ay *(Cheyenne) (from common wheat cv. Cheyenne), *1By9 *and *1Dy10*. The 5' flanking promoter regions of both inactive and active *1Ay *from *T*.*urartu*, *T*.*monococcum aegilopoides*, *T*.*turgidum dicoccon *and *T*. *aestivum *were compared. A few base substitutions and insertions or deletions were found even though the alignment showed high similarity (Figure 4). The N motif, E motif, complete enhancer and TATA box were well conserved in all compared alleles. An 85 bp deletion, in which the partial HMW enhancer was also included, was observed in the 5' flanking promoter regions of all *1Ay *genes from diploid, tetraploid and hexaploid wheats when compared to *1By9 *and *1Dy10 *(Figure 4). Our investigation in the region extended to -845 bp did not find any obvious basis for differential expression. The y-type HMW-GS promoter regions are conserved out to -1200 bp even though some of these genes diverged 4–5 million years ago and the non-coding sequences of wheat diverge fast. Some potential regulatory elements might be in the -845 to -1200 bp region.

**Figure 5:**
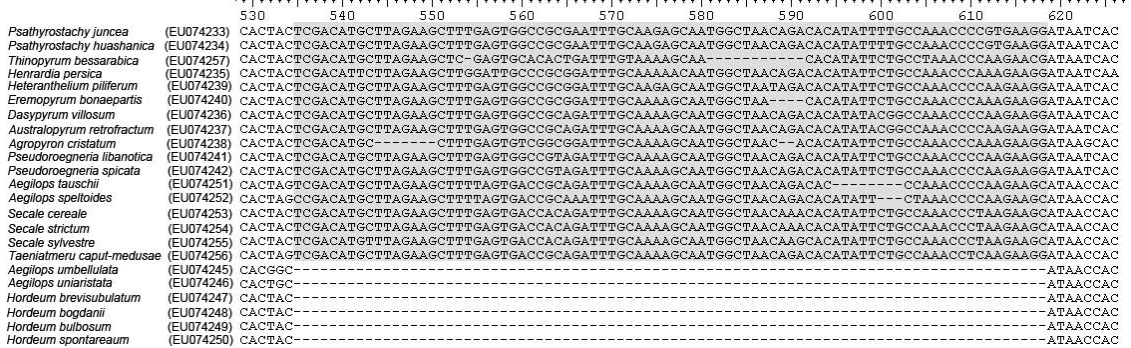
**Comparative analysis of partial 5' flanking region sequences of y-type HMW-GSs from 23 wild diploid relative species of wheat**. The deletion of 85 bp fragment (marked by shadow) was also observed in six diploid species of *Ae*. *umbellulata *(U), *Ae*.* uniaristata *(N), *H*.* bogdanii *(H), *H*.* brevisubulatum *(H), *H*.* bulbosum *(I) and *H*. *spontaneaum *(H).

In order to further understand the control of HMW-GS *1Ay *gene expression, we also characterized the corresponding 5' flanking regions from 23 diploid species of Triticeae. The length of entire 5' flanking regions in 23 Triticeae species varied from 845 to 915 bp (GenBank: EU4233–EU4242, EU4245–EU4257). Multiple sequence alignment showed the 5' flanking of 23 Triticeae species regions were conserved but have more variations than those of *Glu-A1-2 *alleles (Additional file 2). A few substitutions were found in the elements of E motif, N motif, Partial enhancer and Enhancer. Interestingly, the 85 bp deletion was also found in the corresponding regions of y-type HMW-GS and D-hordein genes from six diploid species of *Aegilops umbellulata *(U), *Ae*. *uniaristata *(N), *Hordeum bogdanii *(H), *H*. *brevisubulatum *(H), *H*. *bulbosum *(I) and *H*. *spontareaum *(H) (Figure 5).

### Evolutionary analyses of *Glu-A1-2 *alleles

**Figure 6:**
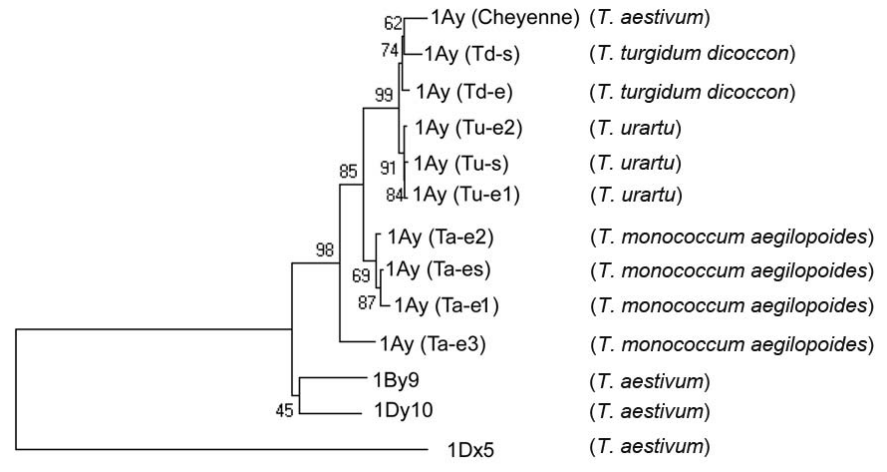
**Phylogenetic relationships of *1Ay *alleles from diploid, tetraploid and hexaploid wheat species with previously published HMW-GS genes encoded by *Glu-B1-2 *and *Glu-D1-2 *loci (represented by *1By9 *and *1Dy10*, respectively)**. The phylogenetic tree was created based on the multiple alignment of the 5' flanking sequences plus the sequences encoding the signal peptides and N-terminal regions. The corresponding sequence of 1Dx5 was used as outgroup, and bootstrap analysis was conducted with 1000 replicates.

The phylogenetic analysis was conducted to investigate the evolutionary relationships among the alleles encoded by *Glu-A1-2*, *Glu-B1-2 *and *Glu-D1-2 *(Figure 6). The 5' flanking sequences plus the sequences encoding the signal peptides and N-terminal domain were chosen to construct the phylogenetic tree under several principles for the sequence selections [36]. Firstly, we found that the regulatory elements that control the tissue specificity and expression level of different HMW-GS genes are well conserved in HMW-GS alleles from 23 diploid species. Secondly, the sequences encoding signal peptides and N-terminal domain are also relative conserved. Therefore, high conservation with enough variations suggested these HMW-GS sequences are phylogenetically informative.

The resulted phylogenetic tree was divided into 2 clusters, comprising the *Glu-A1-2 *alleles at the top and the alleles of *Glu-B1-2 *and *Glu-D1-2 *at the bottom. In the cluster of *Glu-A1-2 *alleles, *1Ay *genes from each species were clustered together, respectively. The *1Ay *genes have been further divided into 3 clusters. *1Ay (Tu-e1)*, *1Ay (Tu-e2)*, *1Ay (Tu-s)*, *1Ay (Td-e)*, *1Ay (Td-s) *and *1Ay (Cheyenne) *were included one groupe showing close relationship; the genes in this group are from *T*. *urartu*, *T*.*turgidum dicoccon *and *T*. *aestivum *respectively. Three genes, *1Ay (Ta-e1)*, *1Ay (Ta-e2) *and *1Ay (Ta-s) *from *T*. *monococcum aegilopoides*, were clustered together while *1Ay (Ta-e3) *was put outside of this cluster. In spite all *1Ay *alleles from different wheats show a close relationship, we noted *1Ay *genes from *T*. *urartu*, *T*.*turgidum dicoccon *and *T*. *aestivum *which were important species involved in wheat evolution, were tightly clustered together in one group; however the *1Ay *genes from *T*. *monococcum aegilopoides *exhibited a more distant relationship to the genes of this group. And this group is supported by high bootstrap values, indicating that strong statistic support for the close relationship of the *Glu-A1-2 *alleles from *T*. *urartu*, *T*.*turgidum dicoccon *and *T*. *aestivum*.

## Discussion

The HMW-GS 1Ay subunits are special because they are always silent in hexaploid wheat. Relative fewer researches have been conducted on this allele when compared to other loci of *Glu-B1 *and *Glu-D1 *[20,32,37]. These informations are not sufficient to understand the expression and heredity of 1Ay subunits. Our investigations on 1Ay alleles with differential expressions would be useful to enhance our knowledge on *Glu-A1-2 *alleles. In genomic PCR, the high fidelity polymerase was used to ensure that the amplified fragments are the accurate representative of interest genes. To avoid potential mistakes introduced by amplification and sequencing, each nucleotide sequence of coding and 5' flanking promoter was determined by using sequencing results of multiple independent clones. Therefore, the molecular information we generated for *1Ay *genes is reliable and effective for exploring structural differentiation and evolution of *Glu-A1 *alleles.

### The structure variations and evolution of *Glu-A1-2 *alleles

Previous genetic researches suggested that there are two tightly linked HMW-GS genes for each genome of wheat and its wild relatives. However, we have only amplified one band representing y-type HMW-GS genes in genomic PCR of *T*.*urartu *and *T*.*monococcum aegilopoides*. Bai et al [32] reported their PCR amplification for HMW-GS ORFs could not obtain x-type genes either. Liu et al. have shown the similar results in the cloning of HMW-GS genes from decaploid *Agropyron elongatum *[30]. Only 15 of 20 genes can be isolated; the rest of 5 x-type ones can not be obtained, and they proposed that the failure in amplification was possibly due to the x-type genes were less conserved or polymeric than y-type ones. In addition, either deletion of sequences/genes or transposon insertions can also prevent the amplification of interest fragments. Therefore, sequence polymorphisms, deletion or transposon insertions may be the reason why we could not obtain the other fragment for x-type amplicon in diploid wheats.

The possession of larger molecular mass and fewer conserved cysteine residues are unique characteristics of 1Ay subunits tested in this study. 1Ay subunits differs from each other and those of known HMW subunits by substitutions, insertions or/and deletions involving single or more amino acid residues (Figure 3). The repetitive domains of 1Ay subunits possess most variations, whereas the N- and C-terminal are relatively conserved only with some substitutions of single amino acid. With a larger molecular weight than other y-type subunits, *1Ay (Ta-e3) *is one of the known largest y-type HMW-GS genes. The unusual large size of 1Ay (Ta-e3) is mainly due to the insertions of repeat units in central repetitive region. Belton [38] and Feeney et al. [39] proposed a model in which the gluten polymers interact via inter-chain hydrogen bonds between the subunit repetitive domains, and more stable interactions can be formed with longer subunits. The positive relationship between the size of HMW-GSs and their effect on dough strength has been reported [40]. The 1Ay (Ta-e3) is longer than other y-type subunits, so we predicted it may have a potential ability to strengthen the gluten polymer interactions.

The y-type HMW-GSs (i.e., *Glu-B1-2 *and *Glu-D1-2 *encoded subunits) in hexaploid wheat have a cysteine residue at the end of repetitive domain. We found that this cysteine residue is not always present in wild wheats. This cysteine residue is replaced by phenylalanine since whose codons, TTT or TTC can be easily converted from cysteine codons TGT or TGC. The number and distribution of cysteine residues in HMW subunit proteins are relevant to their ability to form high molecular polymers stabilized by inter-chain disulphide bonds [41]. In previous report, the substitutions of two cysteine residues in the N-terminal of subunit 1Bx20 resulted in its negative effect on dough strength [42]. This type of cysteine composition in 1Ay subunits has never been reported before our study, it is unknown what would be the effect of the cysteine substitution within repetitive domain to their high order structures. It would be important to express 1Ay subunits in bread wheat to verify their impact to flour quality. In addition, to expressing 1Ay subunit in bread wheat cultivar with 5 native expressed subunits to construct novel transgenic plants which allowed express all 6 x- and y-type HMW-GSs would be considerable interesting.

Because relatively fewer *Glu-A1y *alleles were identified and characterized, the evolution of these alleles has never been reported. Prior to our study, the molecular information on gene structure of *Glu-A1-2 *alleles was only available for *1Ay *from *T*. *urartu *and *T*.*timopheevi *[19,32]. In this study, we are able to investigate the evolution of these alleles based on the identification of novel *1Ay *alleles from more diploid and tetraploid wheats. The close relationship between *1Ay *from *T*. *urartu *and those from *T*.*turgidum dicoccon *and *T*. *aestivum *is supported by phylogenetic analysis and comparison of amino acid sequences; while the *1Ay *genes from *T*. *monococcum aegilopoides *have a little more distant relationship to those of *T*.*turgidum dicoccon *and *T*. *aestivum *(Figure 3 and 6). For the close relationship of these alleles, it may be explained that *T*. *urartu *is generally accepted donor specie of A genome of *T*.*turgidum dicoccon *and *T*. *aestivum *[43,44].

The evolution of new allelic subunit can be formed through the variations of number and distribution of cysteine residues [36]. For example, the good quality subunit 1Dx5 is a novel subunit with an extra cysteine residue in the repetitive domain [3]. In our finding, the conserved cysteine residue at the end of the repetitive domain were all replaced in 1Ay subunits from *T*. *urartu*, *T*.*turgidum dicoccon *and *T*. *aestivum *while this cysteine residue is still in 1Ay subunits of *T*. *monococcum aegilopoides*. We suppose that the substitution of this cysteine residue could be relevant to the evolution of *Glu-A1-2 *alleles. The difference in cysteine residue together with results of phylogenetic analysis and protein sequence comparison further supported that 1Ay genes from *T*. *urartu*, *T*. *turgidum dicoccon *and *T*. *aestivum *are closed, but differed from those of *T*. *monococcum aegilopoides*.

### Gene silencing in *Glu-A1-2*

We focused on both promoter and coding regions to understand the silence of *1Ay *alleles. The development of the primers specific for the 5'flanking promoters of *1Ay *genes made it possible to extend these sequences to cover all recognized elements and compare them. Our investigations in the extended 5' flanking promoter sequences identified a few base substitutions and insertions or deletions among 1Ay alleles with differential expressions. Because these substitutions and insertions or deletions are not specific to active or inactive *1Ay *genes, the correlation between these variations and expression of *1Ay *genes has not been supported.

The deleted fragment of 85 bp contained partial HMW enhancer which is a partial copy of complete HMW enhancer. Halford et al. proposed that the 85 bp deletion was responsible for the silencing of *1Ay *gene [45], whereas Colot et al. reported later that the corresponding fragment of *1Dy12 *was not essential for gene regulation [46]. Prior to our study, it has been observed only in the 5' flanking regions of *1Ay *alleles from *T*. *urartu*, cv. Cheyenne and cv. Chinese spring [13,37,45]. In this study, the 85 bp deletion was also found in the corresponding regions of all *1Ay *genes from *T*. *monococcum aegilopoides *and *T*. *turgidum dicoccon *(Figure 4). Further examinations in the 5' flanking sequences of *Glu-1-2 *alleles from 23 wild diploid wheat species revealed that the 85 bp fragment deletion was also present in 6 species of *Aegilops *and *Hordeum*. Therefore, the 85 bp deletion is not specific for inactive *1Ay *genes. Anderson et al. and Li et al. reported there was a 185 bp insertion in the 5' flanking regions of *1Bx7 *and *1Bx14 *when compared to *1Bx17 *and *1Bx20 *[36,47]. They concluded that this insertion has not disrupted the expression of *1Bx14 *and *1Bx20*. Because the *1Bx *gene of cv. Chinese spring has apparently higher expression than the allelic of cv. Cheyenne *1Bx *gene, the relationship of the *1Bx *promoter *cereal box *duplication to protein synthesis levels was examined. Since both *1Bx *genes contain the same duplication in the promoter, the relationship between different levels of hexaploid *1Bx *genes and the duplication of regulatory elements is not supported [47]. These finding further supported the 85 bp deletion has not disrupted the control of *1Ay *gene expression and is obviously not responsible for the silencing of *1Ay *genes in diploid, tetraploid and hexaploid wheats.

Three silenced genes of *1Ay (Tu-s)*, *1Ay (Ta-s)*, *1Ay (Td-s) *characterized in this study together with *1Ay (Cheyenne)*, showed that their translations were disrupted by the in-frame premature stop codons. It indicated that they were highly unlikely to be expressed as a full length protein. In fact, such information is consistent with our SDS-PAGE results. However, the silencing of *1Ay *gene in cv. Chinese spring is accompanied by the insertion of an 8 kb transposon-like in its coding region [48]. The defects in the coding regions (premature stop codons and insertion of large transposon-like elements) would be possibly responsible for the silencing of the *1Ay *genes in diploid, tetraploid and hexaploid wheats (Table 2). However, the mechanisms of gene expression and silencing are complicated and could involve the interactions of a number of factors, including specific nucleotide sequencing, chromosome rearrangement, and methylation, etc. Some mutational events in more distant distal promoter regions are possible causes for the inactivation of *1Ay *genes; and more distal sequences are necessary to be examined. In addition, the experiments of *1Ay *promoter function in wheat are required to further study the mechanism of the silencing of *Glu-A1-2 *alleles.

**Table 2:** Comparative analysis of the 5' flanking and coding sequence characteristics in *Glu-A1-2 *alleles from diploid, tetraploid and hexaploid wheats.

				Sequence characteristics		
					
HWM-GS alleles	Species	Genome	Gene expression	5'flanking regions	Coding Region	References
*1Ay (Tu-e1)*	*T. urartu*	AA	active	85 bp deletion		This study
*1Ay (Tu-e2)*	*T. urartu*	AA	active	85 bp deletion		This study
*1Ay (Tu-s)*	*T. urartu*	AA	inactive	85 bp deletion	Stop codon	This study
*1Ay (Ta-e1)*	*T. monococcum aegilopoides*	AA	active	85 bp deletion		This study
*1Ay (Ta-e2)*	*T. monococcum aegilopoides*	AA	active	85 bp deletion		This study
*1Ay (Ta-e3)*	*T. monococcum aegilopoides*	AA	active	85 bp deletion		This study
*1Ay (Ta-e)*	*T. monococcum aegilopoides*	AA	inactive	85 bp deletion	Stop codon	This study
*1Ay (Td-e)*	*T. turgidum dicoccon*	AABB	active	85 bp deletion		This study
*1Ay (Td-s)*	*T. turgidum dicoccon*	AABB	inactive	85 bp deletion	Stop codon	This study
*1Ay (Cheyenne)*	*T. aestivum*	AABBDD	inactive	85 bp deletion	Stop codon	Forde et al. (1985)
*1Ay (Chinese spring)*	*T. aestivum*	AABBDD	inactive	85 bp deletion	transposon-like insertion	Harberd et al. (1987)

Both active and inactive *1Ay *genes from *T. urartu*, *T. monococcum aegilopoides*, *T*. *turgidum dicoccon *and *T. aestivum *shared high homology promoter sequences including 85 bp deletions. Nevertheless, the defects (premature stop codons and transposon-like insertion) were found in the coding regions of all silenced *1Ay *genes.

## Conclusion

The possession of larger molecular mass and fewer conserved cysteine residues are unique characteristics of 1Ay subunits tested in this study. Particularly, *1Ay (Ta-e3) *with an unusual large size, is one of known largest y-type HMW-GS gene and may contribute more to the gluten polymers than other known y-type subunits. It is also interested in observing that the conserved cysteine residue within the repetitive domain of the y-type genes of hexaploid wheat is not always present in wild wheats. The *1Ay *genes from *T*. *urartu *have a closer relationship among, *T*.*turgidum dicoccon *and *T*. *aestivum *than those from *T*.*monococcum aegilopoides*. The 85 bp deletions are present not only in the promoter regions of *Glu-A1-2 *alleles with different expressions but also in the corresponding positions of 6 species of *Aegilops *and *Hordeum*. The 85 bp deletion and some variations in the 5'flanking region, have not interrupted expression of *1Ay *genes, whereas the defects in the coding regions (premature stop codons and insertion of large transposon-like element) would be possibly responsible to the silencing of *1Ay *genes. Some mutational events in more distant distal promoter regions might also be the possible cause of the inactivation of *1Ay *gene.

## Methods

### Plant materials

One hundred and forty-one accessions of *T*.*urartu*, *T*. *monococcum aegilopoides, T*. *monococcum monococcum *and *T*. *turgidum dicoccon *were used in SDS-PAGE analysis. All accessions were kindly provided by USDA-ARS . Fifty-three accessions with expressed 1Ay subunits were screened out from 141 accessions, and 6 accessions with expressed 1Ay subunit plus 3 ones without 1Ay subunit were chosen for cloning experiments (Table 3).

**Table 3:** Some accessions of diploid, tetraploid wheat species chosen for further cloning experiments based on the results of SDS-PAGE.

*Species*	*Accession No*.	*Genome*	*HMW-GS composition*						*1Ay alleles*	GenBank *No*.
					
			*Glu-A1*		*Glu-B1*		*Glu-D1*			
					
			x	y	x	y	x	y		
*T. urartu*	PI 428309	AA	+	+					*1Ay (Tu-e1)*	EU984503
*T. urartu*	PI 428318	AA	+	+					*1Ay (Tu-e2)*	EU984504
*T. urartu*	PI 428308	AA	+	-					*1Ay (Tu-s)*	EU984505
*T. monococcum aegilopoides*	PI 428007	AA	+	+					*1Ay (Ta-e1)*	EU984506
*T. monococcum aegilopoides*	PI 277123	AA	+	+					*1Ay (Ta-e2)*	EU984507
*T. monococcum aegilopoides*	PI 306526	AA	+	+					*1Ay (Ta-e3)*	EU984508
*T. monococcum aegilopoides*	PI 427928	AA	+	-					*1Ay (Ta-s)*	EU984509
*T. turgidum dicoccon*	PI 355477	AABB	+	+	+	+			*1Ay (Td-e)*	EU984510
*T. turgidum dicoccon*	PI 355475	AABB	+	-	+	+			*1Ay (Td-s)*	EU984511
*T. aestivum *cv. Chinese spring		AABBDD	-	-	7	8	2	12		

Plus (+) and minus (-) signs indicate the presence or the absence of the corresponding HMW glutenin subunit, respectively.

### SDS-PAGE

HMW-GSs of *T*. *urartu*, *T*. *monococcum aegilopoides *and *T*.*turgidum dicoccon *were extracted from single half seed according to Mackie et al [49]. SDS-PAGE was conducted as described in Wan et al. [19]. HMW-GSs from hexaploid wheat cv. Chinese Spring (null, 1Bx7+1By8, 1Dx2+1Dy12) were used as references.

### Characterization of the complete ORFs of *1Ay *from diploid and tetraploid wheats

CTAB method was carried out to extract genomic DNA from the leaves of two-week-old single plant [50]. For amplifying the complete coding sequence of *1Ay*, a pair of primers, P1 (5'-ATGGCTAAGCGGC/TTA/GGTCCTCTTTG-3') and P2 (5'-CTATCACTGGCTG/AGCCGACAATGCG-3'), were designed according to the nucleotide sequences conserved in the 5' or 3' ends of the ORFs of published HMW-GSs. The *LA Taq *polymerase (TaKaRa) with GC buffer for GC-rich template was used in the PCR amplification to avoid introducing errors into the sequence. The cycling parameters was 94°C for 5 min, followed by 30 cycles of 94°C for 40 sec, 68°C for 5 min, and a final extension step at 68°C for 15 min[51]. PCR products were separated in 1% agarose gels and all DNA fragments were recovered and purified from agarose gels, and ligated into the pMD18-T vector (TaKaRa). Then the ligation mixtures were transformed into *Escherichia coli *DH5α competent cells. To obtain the full-length sequence, the strategy of primer walking and the nest deletion method according to Sambrook et al. [52] were used. The sequencing was performed by Invitrogen Company (Shanghai, China). The final nucleotide sequences for each ORF of *1Ay *were determined from the sequencing results of 3 independent clones.

### Isolations of the 5' flanking promoter region of *1Ay *genes

Based on the alignment of the sequences of published HMW glutenin genes *1Ax1 *(GenBank: X61009), 1Ax2* (GenBank: M22208), 1Bx7 (GenBank: X13927), 1Bx17 (GenBank: JC2099), 1Dx2(GenBank: X03346), 1Dx5 (GenBank: X12928), 1Ay (GenBank: X03042) 1By9 (GenBank: X61026), 1Dy10 (GenBank: X12929), 1Dy12 (GenBank: X03041) and 1Dy12.1^t ^(GenBank: AY248704), a pair of primers (P3 and P4) specific for the promoter region of *1Ay *was designed. The P3 primer (5'-AGGGAAAGACAATGGACATG-3') was designed from the sequence which was strictly conserved in the 5' flanking regions of all *Glu-1 *loci, whereas the P4 primer (5'-CATCTGGAGCCCCGTGCTC-3') was derived from the sequence coding for 6 amino acid residues (STGLQM) which existed only in y-type HMW-GSs. The amplification profile was 94°C for 5 min, followed by 35 cycles of 94°C for 40 sec, 60°C for 1 min, and 72°C for 1 min 30 sec, and a final extension step at 72°C for 7 min. PCR products were purified, cloned into pMD18-T, and sequenced. The final nucleotide sequences for *1Ay *promoter were also constructed on sequencing at least 3 independent clones.

### Further investigation of the corresponding 5' flanking promoter regions of 23 different diploid wheat species

When carrying out the present studies, we found that all the 5' flanking regions of both inactive and active *1Ay *genes from *T*. *urartu*, *T*. *monococcum aegilopoides*, *T*. *turgidum dicoccon *and *T*.*aestivum*, shared an 85 bp deletion. This deletion has only been identified in the 5 flanking regions of *Glu-A1-2 *alleles but not in any other locus. To ensure the 85 bp deletion was either specific for *Glu-A1-2 *alleles or also present in other alleles, we focused on the corresponding regions of *Glu-1-2 *of other diploid species of Triticeae. It will be helpful to understand the relationship between HMW-GS gene expression and their 5' flanking sequence variations. Then, the 5' flanking sequences of *Glu-1-2 *were characterized by using primers P3 and P4 from 23 diploid species of Triticeae. The PCR amplification, cloning and sequencing were the same *1Ay *promoter characterization mentioned above.

### Nucleotides and protein sequence analyses and evolutionary relationship investigations

The translation of nucleotide sequences was performed by DNAman software package (V5. 2. 10; Lynnon Biosoft). Multiple alignments were carried out with Clustal W (V1.83) for comparisons of DNA or protein sequences [53]. The alignment was further improved by visual examination and manual adjustment. To investigate the phylogenetic relationship of *1Ay *genes from different wheat species with previously characterized *Glu-1-2 *alleles (represented by *1By9 *and *1Dy10*), we selected the nucleotide sequences of the 5'flanking region plus the sequences encoding signal peptides and N-terminal domain (the corresponding region of *1Dx5 *was used as outgroup) to create a multiple alignment by the Clustal W program. The software MEGA 4.02 was used to create phylogenetic trees by neighbour-joining (NJ) method [54].

## Authors' contributions

JQT contributed to design and carry out the experiments and wrote the paper; WYM did the cloning of HWM glutenin ORFs, and revised the manuscript; WF made contribution to SDS-PAGE analysis and promoter cloning of wild diploid species; WJR and YZH did the analysis of the data; ZYL contributed to improve research programme and review the manuscript. All authors have read and approved the final manuscript.

## Supplementary Material

Additional File 1 **The summary of HMW-GS composition of 141 accessions from diploid and tetraploid wheats, identified by SDS-PAGE.** Plus (+) and minus (-) signs indicate the presence or the absence of the corresponding HMW glutenin subunit, respectively. The expression frequency of 1Ay subunits is showed as percent, and the numbers in bracket represent the ratio of accessions with expressed 1Ay subunits to the total. Click here for file

Additional File 2 **Full alignment of promoter sequences of 23 species of Triticeae.** The regulatory elements were labelled and indicated by box, respectively. TATA box was indicated by asterisks. The 85-bp fragment deletions were marked by shadow. Click here for file
