# Supplementary material for: Characterization and comparative analysis of HMW glutenin 1Ay alleles with differential expressions
Source: BMC Plant Biol. 2009 Feb 6;9:16. doi: 10.1186/1471-2229-9-16 (PMC2667398; doi:10.1186/1471-2229-9-16)
Supplement: Additional File 1 — The summary of HMW-GS composition of 141 accessions from diploid and tetraploid wheats, identified by SDS-PAGE. Plus (+) and minus (-) signs indicate the presence or the absence of the corresponding HMW glutenin subunit, respectively. The expression frequency of 1Ay subunits is showed as percent, and the numbers in bracket represent the ratio of accessions with expressed 1Ay subunits to the total. [file 1471-2229-9-16-S1.pdf]

**Additional file 1.** The summary of HMW-GS composition of 141 accessions from diploid and tetraploid wheats, identified by SDS-PAGE analysis. Plus (+) and minus (-) signs indicate the presence or the absence of the corresponding HMW glutenin subunit, respectively. The expression frequency of 1Ay subunits is showed as percent, and the numbers in bracket represent the ratio of expressed 1Ay subunits to the total.

| Species | Accession NO. | Genome | HMW-GS composition | | | | Expression frequency of 1Ay subunits in each wheat |
|---|---|---|---|---|---|---|---|
| | | | 1Ax | 1Ay | 1Bx | 1By | |
| *Triticum monococcum subsp. aegilopoides* | CItr 17665 | AA | + | + | | | |
| *Triticum baeoticum Boiss* | PI 277123 | AA | + | + | | | |
| *Triticum monococcum subsp. aegilopoides* | PI 306526 | AA | + | + | | | |
| *Triticum monococcum subsp. aegilopoides* | PI 352502 | AA | + | + | | | |
| *Triticum monococcum subsp. aegilopoides* | PI 374574 | AA | + | - | | | |
| *Triticum monococcum subsp. aegilopoides* | PI 427405 | AA | + | + | | | |
| *Triticum monococcum subsp. aegilopoides* | PI 427447 | AA | + | - | | | |
| *Triticum monococcum subsp. aegilopoides* | PI 427603 | AA | + | - | | | |
| *Triticum monococcum subsp. aegilopoides* | PI 427604 | AA | + | - | | | |
| *Triticum monococcum subsp. aegilopoides* | PI 427605 | AA | + | - | | | |
| *Triticum monococcum subsp. aegilopoides* | PI 427607 | AA | + | - | | | |
| *Triticum monococcum subsp. aegilopoides* | PI 427613 | AA | + | - | | | |
| *Triticum monococcum subsp. aegilopoides* | PI 427621 | AA | + | + | | | |
| *Triticum monococcum subsp. aegilopoides* | PI 427622 | AA | + | - | | | |
| *Triticum monococcum subsp. aegilopoides* | PI 427680 | AA | + | - | | | |
| *Triticum monococcum subsp. aegilopoides* | PI 427754 | AA | + | + | | | |
| *Triticum monococcum subsp. aegilopoides* | PI 427755 | AA | + | - | | | |
| *Triticum monococcum subsp. aegilopoides* | PI 427759 | AA | + | + | | | |
| *Triticum monococcum subsp. aegilopoides* | PI 427967 | AA | + | + | | | |
| *Triticum monococcum subsp. aegilopoides* | PI 538510 | AA | + | + | | | |
| *Triticum monococcum subsp. aegilopoides* | PI 538584 | AA | + | + | | | |
| *Triticum monococcum subsp. aegilopoides* | PI 538585 | AA | + | + | | | |
| *Triticum monococcum subsp. aegilopoides* | PI 538586 | AA | + | + | | | |
| *Triticum monococcum subsp. aegilopoides* | PI 538720 | AA | + | + | | | |
| *Triticum monococcum subsp. aegilopoides* | PI 538723 | AA | + | - | | | |
| *Triticum monococcum subsp. aegilopoides* | PI 542475 | AA | + | + | | | |
| *Triticum monococcum subsp. aegilopoides* | PI 560865 | AA | + | + | | | |
| *Triticum monococcum subsp. aegilopoides* | PI 560866 | AA | + | - | | | |
| *Triticum monococcum subsp. aegilopoides* | PI 560869 | AA | + | + | | | |
| *Triticum monococcum subsp. aegilopoides* | PI 614650 | AA | + | + | | | 60% (18/30) |
| *Triticum urartu* | PI 428308 | AA | + | - | | | |
| *Triticum urartu* | PI 428309 | AA | + | + | | | |
| *Triticum urartu* | PI 428310 | AA | + | - | | | |
| *Triticum urartu* | PI 428311 | AA | + | + | | | |
| *Triticum urartu* | PI 428312 | AA | + | + | | | |
| *Triticum urartu* | PI 428313 | AA | + | + | | | |
| *Triticum urartu* | PI 428314 | AA | + | + | | | |
| *Triticum urartu* | PI 428315 | AA | + | + | | | |
| *Triticum urartu* | PI 428316 | AA | + | + | | | |
| *Triticum urartu* | PI 428317 | AA | + | + | | | |
| *Triticum urartu* | PI 428318 | AA | + | + | | | |

| | | | | | | | |
|---|---|---|---|---|---|---|---|
| *Triticum urartu* | PI 428319 | AA | + | + | | | |
| *Triticum urartu* | PI 428320 | AA | + | + | | | |
| *Triticum urartu* | PI 428321 | AA | + | + | | | |
| *Triticum urartu* | PI 428322 | AA | + | + | | | |
| *Triticum urartu* | PI 428323 | AA | + | + | | | |
| *Triticum urartu* | PI 428324 | AA | + | + | | | 87.5% (15/17) |
| *Triticum monococcum subsp. monococcum* | PI 272535 | AA | + | + | | | |
| *Triticum monococcum subsp. monococcum* | PI 272563 | AA | + | + | | | |
| *Triticum monococcum subsp. monococcum* | PI 286068 | AA | + | + | | | |
| *Triticum monococcum subsp. monococcum* | PI 289599 | AA | + | + | | | |
| *Triticum monococcum subsp. monococcum* | PI 355528 | AA | + | + | | | |
| *Triticum monococcum subsp. monococcum* | PI 355529 | AA | + | + | | | |
| *Triticum monococcum subsp. monococcum* | PI 355539 | AA | + | - | | | 85.7% (6/7) |
| *Triticum turgidum subsp. dicoccoides* | PI 346783 | AABB | + | - | + | + | |
| *Triticum turgidum subsp. dicoccoides* | PI 428035 | AABB | + | + | + | + | |
| *Triticum turgidum subsp. dicoccoides* | PI 428042 | AABB | + | - | + | + | |
| *Triticum turgidum subsp. dicoccoides* | PI 428109 | AABB | + | - | + | - | |
| *Triticum turgidum subsp. dicoccoides* | PI 428133 | AABB | + | + | + | + | |
| *Triticum turgidum subsp. dicoccoides* | PI 428134 | AABB | + | - | + | + | |
| *Triticum turgidum subsp. dicoccoides* | PI 428136 | AABB | + | + | + | + | |
| *Triticum turgidum subsp. dicoccoides* | PI 428137 | AABB | + | - | + | + | |
| *Triticum turgidum subsp. dicoccoides* | PI 466964 | AABB | + | - | + | + | |
| *Triticum turgidum subsp. dicoccoides* | PI 466967 | AABB | + | - | + | + | |
| *Triticum turgidum subsp. dicoccoides* | PI 466968 | AABB | + | + | + | + | |
| *Triticum turgidum subsp. dicoccoides* | PI 466969 | AABB | + | - | + | + | |
| *Triticum turgidum subsp. dicoccoides* | PI 466973 | AABB | + | + | + | + | |
| *Triticum turgidum subsp. dicoccoides* | PI 466975 | AABB | + | + | + | + | |
| *Triticum turgidum subsp. dicoccoides* | PI 466976 | AABB | + | + | + | + | |
| *Triticum turgidum subsp. dicoccoides* | PI 470980 | AABB | + | - | + | + | |
| *Triticum turgidum subsp. dicoccoides* | PI 470981 | AABB | + | + | + | + | |
| *Triticum turgidum subsp. dicoccoides* | PI 470982 | AABB | + | - | + | + | |
| *Triticum turgidum subsp. dicoccoides* | PI 470983 | AABB | + | - | + | + | |
| *Triticum turgidum subsp. dicoccoides* | PI 481504 | AABB | + | - | + | - | |
| *Triticum turgidum subsp. dicoccoides* | PI 481505 | AABB | - | - | + | - | |
| *Triticum turgidum subsp. dicoccoides* | PI 481506 | AABB | + | + | + | + | |
| *Triticum turgidum subsp. dicoccoides* | PI 487258 | AABB | + | - | + | + | |
| *Triticum turgidum subsp. dicoccoides* | PI 487260 | AABB | + | - | + | + | |
| *Triticum turgidum subsp. dicoccoides* | PI 487262 | AABB | + | - | + | - | |
| *Triticum turgidum subsp. dicoccoides* | PI 487263 | AABB | + | - | + | - | |
| *Triticum turgidum subsp. dicoccoides* | PI 487264 | AABB | + | - | + | + | |
| *Triticum turgidum subsp. dicoccoides* | PI 503312 | AABB | + | - | + | - | |
| *Triticum turgidum subsp. dicoccoides* | PI 503313 | AABB | + | - | + | - | |
| *Triticum turgidum subsp. dicoccoides* | PI 554584 | AABB | + | - | + | + | 30%(9/30) |
| *Triticum turgidum subsp. dicoccon* | PI 182743 | AABB | + | - | + | + | |

| | | | | | | | |
|---|---|---|---|---|---|---|---|
| *Triticum turgidum subsp. dicoccon* | PI 191091 | AABB | + | - | + | + | |
| *Triticum turgidum subsp. dicoccon* | PI 306534 | AABB | + | - | + | + | |
| *Triticum turgidum subsp. dicoccon* | PI 306536 | AABB | + | - | + | + | |
| *Triticum turgidum subsp. dicoccon* | PI 306538 | AABB | + | - | + | - | |
| *Triticum turgidum subsp. dicoccon* | PI 306539 | AABB | + | - | + | + | |
| *Triticum turgidum subsp. dicoccon* | PI 308879 | AABB | + | - | + | + | |
| *Triticum turgidum subsp. dicoccon* | PI 319868 | AABB | - | - | + | + | |
| *Triticum turgidum subsp. dicoccon* | PI 355466 | AABB | + | + | + | + | |
| *Triticum turgidum subsp. dicoccon* | PI 355467 | AABB | + | - | + | - | |
| *Triticum turgidum subsp. dicoccon* | PI 355475 | AABB | + | - | + | + | |
| *Triticum turgidum subsp. dicoccon* | PI 355477 | AABB | + | + | + | + | |
| *Triticum turgidum subsp. dicoccon* | PI 355479 | AABB | + | - | + | + | |
| *Triticum turgidum subsp. dicoccon* | PI 355480 | AABB | + | - | + | + | |
| *Triticum turgidum subsp. dicoccon* | PI 387754 | AABB | - | - | + | + | |
| *Triticum turgidum subsp. dicoccon* | PI 479964 | AABB | - | - | + | + | |
| *Triticum turgidum subsp. dicoccon* | PI 479966 | AABB | - | - | + | + | |
| *Triticum turgidum subsp. dicoccon* | PI 480068 | AABB | + | - | + | - | |
| *Triticum turgidum subsp. dicoccon* | PI 480313 | AABB | - | - | + | + | 10.5% (2/19) |
| *Triticum turgidum subsp. durum* | CItr 3323 | AABB | + | - | - | + | |
| *Triticum turgidum subsp. durum* | CItr 2086 | AABB | + | - | - | + | |
| *Triticum turgidum subsp. durum* | PI 88737 | AABB | - | - | + | - | |
| *Triticum turgidum subsp. durum* | PI 91672 | AABB | + | - | + | - | |
| *Triticum turgidum subsp. durum* | PI 91673 | AABB | + | - | + | - | |
| *Triticum turgidum subsp. durum* | PI 109585 | AABB | + | - | + | - | |
| *Triticum turgidum subsp. durum* | PI 109588 | AABB | - | - | + | - | |
| *Triticum turgidum subsp. durum* | PI 109589 | AABB | + | - | + | - | |
| *Triticum turgidum subsp. durum* | PI 109593 | AABB | - | - | + | - | |
| *Triticum turgidum subsp. durum* | PI 109597 | AABB | - | - | + | - | |
| *Triticum turgidum subsp. durum* | PI 133459 | AABB | + | - | + | + | |
| *Triticum turgidum subsp. durum* | PI 220426 | AABB | + | - | + | + | |
| *Triticum turgidum subsp. durum* | PI 225167 | AABB | - | - | + | - | |
| *Triticum turgidum subsp. durum* | PI 254191 | AABB | + | - | + | - | |
| *Triticum turgidum subsp. durum* | PI 265017 | AABB | + | - | + | + | |
| *Triticum turgidum subsp. durum* | PI 278553 | AABB | - | - | + | + | |
| *Triticum turgidum subsp. turgidum* | PI 289822 | AABB | - | + | + | + | |
| *Triticum turgidum subsp. durum* | PI 308878 | AABB | + | + | + | + | |
| *Triticum turgidum subsp. durum* | PI 352353 | AABB | + | - | + | - | |
| *Triticum aestivum subsp. aestivum* | PI 428649 | AABB | + | + | + | + | |
| *Triticum turgidum subsp. durum* | PI 429317 | AABB | - | - | + | + | |
| *Triticum turgidum subsp. durum* | PI 430748 | AABB | - | - | + | + | |
| *Triticum turgidum subsp. durum* | PI 480458 | AABB | + | - | + | - | |
| *Triticum turgidum subsp. durum* | PI 480459 | AABB | + | - | + | - | |
| *Triticum turgidum subsp. durum* | PI 480463 | AABB | - | - | + | + | |
| *Triticum turgidum subsp. durum* | PI 496260 | AABB | + | - | + | + | |

| | | | | | | | |
|---|---|---|---|---|---|---|---|
| *Triticum turgidum subsp. durum* | PI 519864 | AABB | + | - | + | - | |
| *Triticum turgidum subsp. durum* | PI 520393 | AABB | - | - | + | + | |
| *Triticum turgidum subsp. durum* | PI 520394 | AABB | - | - | + | + | |
| *Triticum turgidum subsp. durum* | PI 520399 | AABB | - | - | + | + | |
| *Triticum turgidum subsp. durum* | PI 520415 | AABB | - | - | + | - | |
| *Triticum turgidum subsp. durum* | PI 583728 | AABB | - | - | + | - | |
| *Triticum turgidum subsp. durum* | PI 591064 | AABB | + | - | + | - | |
| *Triticum turgidum subsp. durum* | PI 591065 | AABB | + | - | + | - | |
| *Triticum turgidum subsp. durum* | PI 593002 | AABB | + | - | + | - | |
| *Triticum turgidum subsp. durum* | PI 593003 | AABB | + | - | + | - | |
| *Triticum turgidum subsp. durum* | PI 593005 | AABB | - | - | + | - | |
| *Triticum turgidum subsp. durum* | PI 593006 | AABB | + | - | + | - | 7.8% (3/38) |
| Total | 141 | | | 53 | | | 37.6%(53/141) |
