# Supplementary figures and images for: Characterization and comparative analysis of HMW glutenin 1Ay alleles with differential expressions
Source: BMC Plant Biol. 2009 Feb 6;9:16. doi: 10.1186/1471-2229-9-16 (PMC2667398; doi:10.1186/1471-2229-9-16)

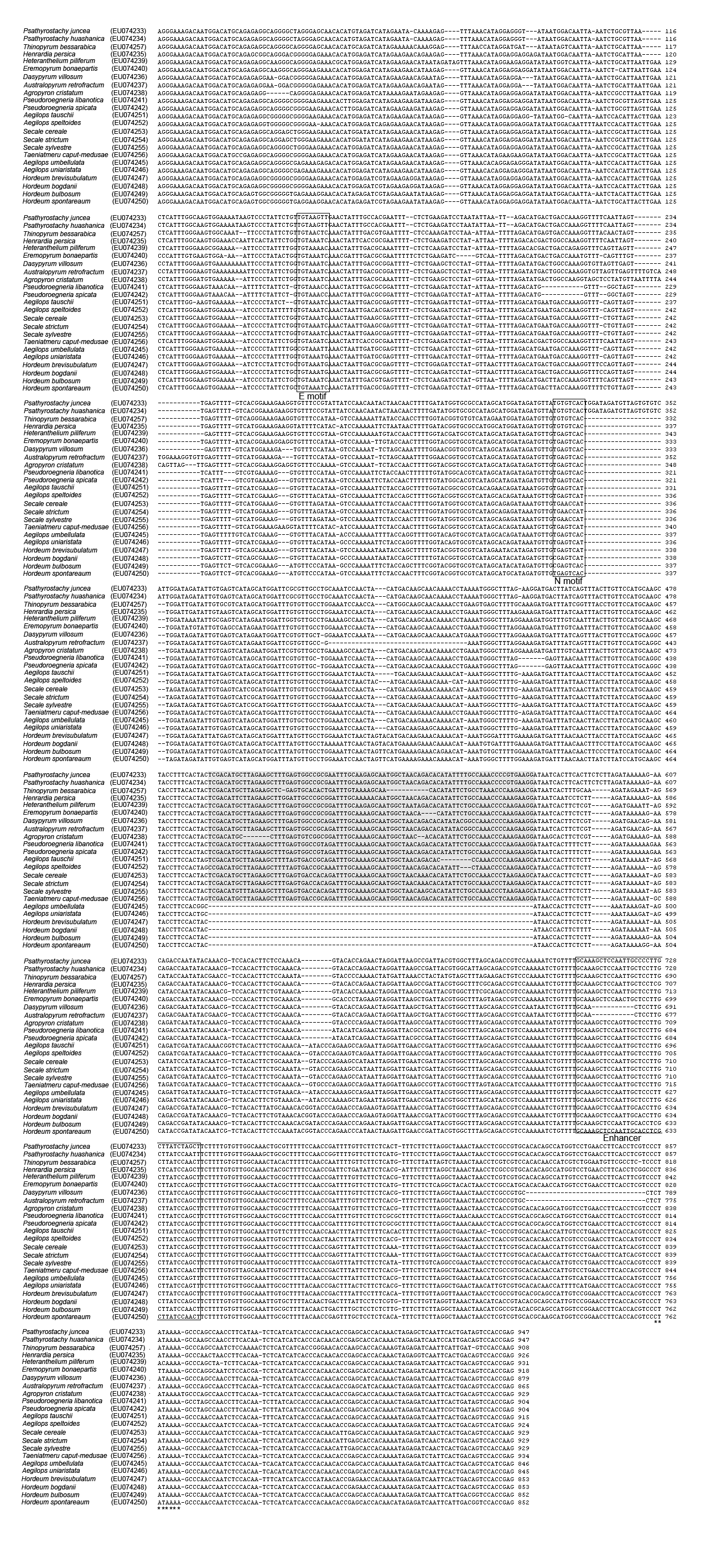

Supplement: Additional File 2 — Full alignment of promoter sequences of 23 species of Triticeae. The regulatory elements were labelled and indicated by box, respectively. TATA box was indicated by asterisks. The 85-bp fragment deletions were marked by shadow. [file 1471-2229-9-16-S2.tiff]
